# Integrated analysis of transcriptomic and metabolomic data demonstrates the significant role of pyruvate carboxylase in the progression of ovarian cancer

**DOI:** 10.18632/aging.104004

**Published:** 2020-11-07

**Authors:** Hongkai Shang, Jianfeng Zheng, Jinyi Tong

**Affiliations:** 1 Department of Gynecology, Affiliated Hangzhou First People's Hospital, Zhejiang University School of Medicine, Hangzhou 310006, Zhejiang Province, China; 2 Department of Gynecology, Affiliated Hangzhou First People's Hospital, Nanjing Medical University, Hangzhou 310006, Zhejiang Province, China

**Keywords:** epithelial ovarian cancer, differentially expressed genes, prognosis, transcriptome sequencing, metabolomics sequencing

## Abstract

The aim of this study was to explore prognosis-related biomarkers and underlying mechanisms during ovarian carcinoma progression and development. mRNA expression profiles and GSE49997 dataset were downloaded. Survival analyses were performed for genes with high expression levels. Expression level of candidate genes was explored in four ovarian cancer cells lines. Pyruvate carboxylase (PC) was found to be one of significantly differentially expressed gene (DEG). The role of *PC* knockdown was analyzed in SKOV cells using cell proliferation, flow cytometric, and Transwell migration and invasion assays. DEGs and metabolites in *PC*-shRNA (shPC)-treated samples vs. control groups were identified. *PC* was a prognosis-related gene and related to metabolic pathway. Knockdown of *PC* regulated cell proliferation, cell cycle progression, and migration and invasion of SKOV-3 cells. Transcriptome sequencing analyses showed *STAT1* and *TP53* gained higher degrees in PPI network. A total of 44 metabolites were identified. These DEGs and metabolites in PC samples were related with neuroactive ligands receptor interaction, glycine, serine and threonine metabolism, and ABC transporter pathways. PC may affect the tumor biology of ovarian cancer through the dysregulation of glycine, serine, and threonine metabolism, and ABC transporter pathways, as well as *STAT1* and *TP53* expression.

## INTRODUCTION

Ovarian cancer is 9^th^ common malignancies in women worldwide and 8^th^ most common cause of cancer death. According to the GLOBOCAN 2018 database, there are approximately 295,414 new cases and 184,799 deaths of ovarian cancer [1]. There are multiple risk factors in the development of ovarian cancer, such as increased ovulation over their lifetime, hormone therapy after menopause, fertility medication, and obesity [2–4]. Epithelial ovarian carcinoma (EOC) is the most common type of ovarian cancer accounting for 90% of all ovarian cancer cases [5] and a major cause of death from gynecologic cancers [6]. Ovarian cancer is usually asymptomatic in the early stages and typically diagnosed in the advanced stage, which makes treatment more challenging. Furthermore, EOC is prone to recur and migrate, which often leads to poor prognosis.

Currently, surgery and cytotoxic chemotherapy can effectively improve clinical outcomes and are indicated as primary therapies for patients with ovarian cancer; however, these treatment modalities often fail to cure the disease at terminal stages [7]. The standard treatment for advanced ovarian cancer is aggressive cytoreductive surgery combined with platinum and taxane-based chemotherapy. Despite several advances in the treatment of ovarian cancer, the survival rate is discouragingly low in recurrent chemo-resistant ovarian cancers [8]. Therefore, it is important to discover new biomarkers for improving the diagnosis and prognosis of patients with ovarian cancer. Metabolomics has been widely used to discover key molecular changes underlying disease pathophysiology [9, 10]. Using integrated analyses of metabolomic and transcriptomic data, researchers can greatly increase the understanding of metabolic networks and biological systems. Recently, a series of studies uncovered potential biomarkers and biological processes in several cancer types using an integrated analysis of metabolomic and transcriptomic data [11–13]. Susan et al. demonstrated that the metabolism of branched-chain amino acids (BCAAs) was altered in type 2 diabetes (T2D), caused by reduced catabolism, and proposed this alteration as a novel biomarker for T2D [14]. An additional study showed that dysregulation of the lipolytic pathway, involving lipases, contributes to the development of pancreatic cancer, and several saturated free fatty acids (FFAs) were closely related to the proliferation of pancreatic cancer cells [15]. However, integrated analyses of metabolomics and transcriptomics data in biomarker and pathway discovery for ovarian cancers are rare. In this study, we aimed to identify potential and reliable biomarkers with prognostic value using an integrated analysis of metabolomic and transcriptomics data in ovarian cancer. According to preliminary bioinformatic analyses, pyruvate carboxylase (*PC*) was found to be a prognosis-related gene significantly enriched in the metabolic pathway. The potential role of PC in ovarian cancer development was investigated by assaying cell proliferation, invasion, and metastasis in ovarian tumor cells. Related mechanisms were explored using an integrated analysis of transcriptomic and metabolomics data. Our study demonstrates a promising approach to investigate the metabolic mechanism of ovarian cancer with the aim of discovering more reliable biomarkers with prognostic value.

## RESULTS

### Identifying candidate genes significantly associated with survival of EOC

To identify the candidate genes with prognostic value in ovarian cancer, we downloaded the gene expression profiles from the TCGA and GEO databases, which was followed by screening of DEGs and text mining.

**Figure 1:**
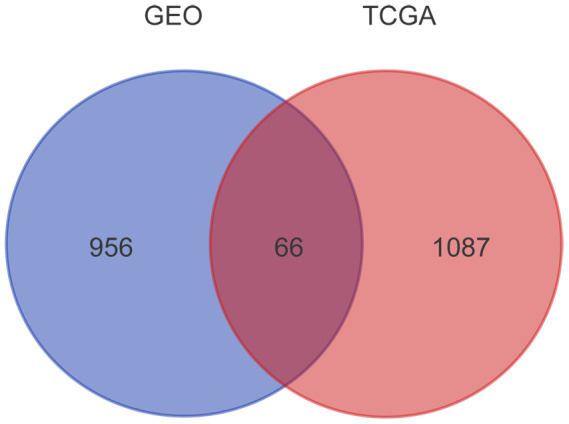
**Prognosis-related genes screened from TCGA and GSE49997 datasets.** The gene expression profiles from TCGA and GEO databases were subjected to survival analyses. A total of 1153 and 1022 genes related with prognosis were obtained from TCGA and GSE49997 datasets, respectively. After Venn diagram analysis, 66 overlapping genes were identified as candidate genes related to EOC progression.

Overall, we screened 1153 and 1022 genes related with prognosis from TCGA and GSE49997 dataset, respectively. After Venn diagram analysis, 66 overlapping genes were identified as candidate genes related to EOC progression (Figure 1).

**Figure 2:**
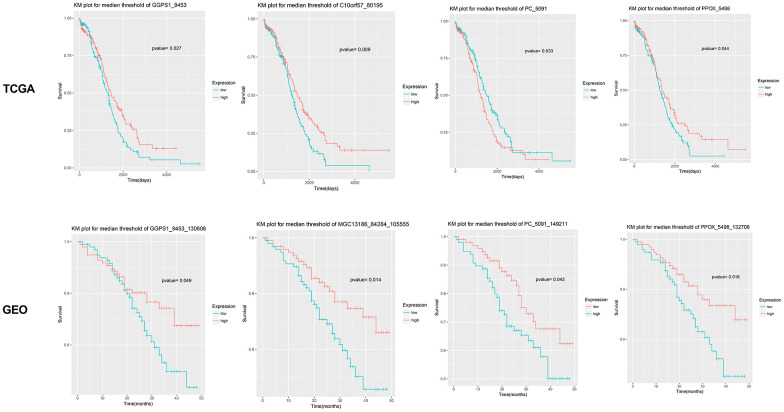
**Survival curve for four candidate genes closely related with the prognosis of epithelial ovarian cancer.** Results show that *GGPS1*, *NTPCR* (also known as *C1orf57* or *MGC13186*), *PC*, and *PPOX* are significantly associated with the prognosis of epithelial ovarian cancer. The survival curves based on TCGA and GSE49997 datasets are listed, separately.

After text mining, a total of 16 genes were found to be associated with EOC (Table 1). KEGG pathway analyses showed that the metabolic pathway (hsa01100) was the most significant pathway involved with *GGPS1, ME1, DSE, NTPCR* (also known as *C1orf57, MGC13186*), *PPOX,* and *PC* (Table 2)*.* Among the metabolic pathway-related genes, *ME1* and *DSE* have been reported to be associated with ovarian cancer. The KM survival curve for the remaining four genes is shown in Figure 2. High expression levels of *GGPS1* and *NTPCR* correlated with a longer patient survival time, while high expression levels of *PC* and *PPOX* were closely related to a shorter patient-survival time.

**Table 1:** Text mining analysis for 16 genes in PubMed dataset associated with ovarian cancer.

**Gene**	**count**	**PubMed ID**
**FOXA2**	8	pmid28965696|pmid28393191|pmid27538367|pmid27430660|pmid26512061|pmid25715123|pmid22172062|pmid20066894|pmid
**KDM3A**	5	pmid28925393|pmid28521455|pmid27694900|pmid26779649|pmid26498640|
**BCHE**	5	pmid28350120|pmid27465871|pmid26886260|pmid17192624|pmid7896186|
**ME1**	3	PMID: 8888953|PMID: 2305835 |PMID: 3276635
**CXCL14**	3	pmid28087599|pmid26893359|pmid24700803|
**NUAK1**	3	pmid27833898|pmid26151663|pmid24700803|
**WDR77**	2	pmid28407786|pmid22022581|
**DSE**	2	PMID: 11092984|PMID: 4028011
**ABCA3**	2	pmid24814220|pmid14973057|
**BCL2L2**	1	PMID: 14973057
**h2afy**	1	pmid22589551|
**lpar6**	1	pmid24994816|
**FREM2**	1	pmid23707566|
**ASAP3**	1	pmid26886260|
**OTUB2**	1	pmid17961127|
**SSBP1**	1	pmid20969748|

**Table 2:** Functional enrichment analysis for candidate genes screened from TCGA-ovarian cancer and GSE49997 datasets.

**Term**	**Count**	**Genes**	**P values**
**hsa01100:Metabolic pathways**	6	GGPS1;ME1;DSE;NTPCR;PPOX;PC	0.00465229
**hsa04924:Renin secretion**	3	ACE;CACNA1C;PPP3CA	0.022092
**hsa04310:Wnt signaling pathway**	3	PRICKLE2;TCF7L1;PPP3CA	0.0088142
**hsa05200:Pathways in cancer**	3	lpar6;TCF7L1;CSF3R	0.0420139
**hsa00620:Pyruvate metabolism**	2	ME1;PC	0.0135316
**hsa02010:ABC transporters**	2	ABCA3;ABCB9	0.0147837
**hsa05031:Amphetamine addiction**	2	CACNA1C;PPP3CA	0.0213649
**hsa04720:Long-term potentiation**	2	CACNA1C;PPP3CA	0.0213649
**hsa05412:Arrhythmogenic right ventricular cardiomyopathy (ARVC)**	2	TCF7L1;CACNA1C	0.022791
**hsa05410:Hypertrophic cardiomyopathy (HCM)**	2	ACE;CACNA1C	0.024746
**hsa01200:Carbon metabolism**	2	ME1;PC	0.0338292
**hsa04724:Glutamatergic synapse**	2	CACNA1C;PPP3CA	0.0340726
**hsa04142:Lysosome**	2	ABCB9;dnase2	0.0357524
**hsa04728:Dopaminergic synapse**	2	CACNA1C;PPP3CA	0.037391
**hsa04921:Oxytocin signaling pathway**	2	CACNA1C;PPP3CA	0.0439704
**hsa04022:cGMP-PKG signaling pathway**	2	CACNA1C;PPP3CA	0.0456096
**hsa05010:Alzheimer's disease**	2	CACNA1C;PPP3CA	0.0460121
**hsa04020:Calcium signaling pathway**	2	CACNA1C;PPP3CA	0.0481754
**hsa04060:Cytokine-cytokine receptor interaction**	2	CSF3R;CXCL14	0.00571639
**hsa04010:MAPK signaling pathway**	2	CACNA1C;PPP3CA	0.00610035
**hsa04151:PI3K-Akt signaling pathway**	2	lpar6;CSF3R	0.00722717

### The dysregulation of metabolic pathway-related genes in ovarian cancer cells

**Figure 3:**
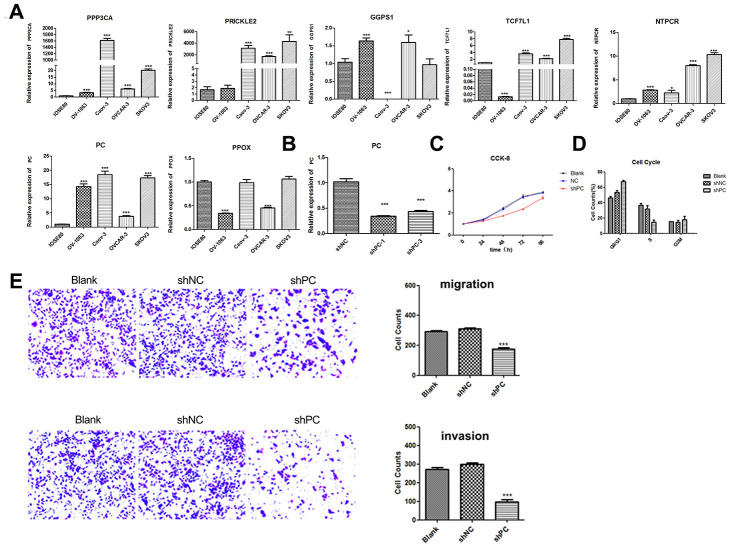
**Knockdown of *PC* can significantly inhibit cell proliferation, cell cycle progression, and cell migration and invasion.** (**A**) The relative expression of *GGPS1*, *NTPCR*, *PPOX*, *PC*, *PRICKLE2*, *TCF7L1,* and *PPP3CA* in four cancer cell lines (SKOV3, CAOV-3, OV-1063, and OVCAR-3). Gene expression levels of candidate genes (*GGPS1*, *NTPCR*, *PPOX*, *PC*, *PRICKLE2*, *TCF7L1,* and *PPP3CA*) were examined in different ovarian cancer cells lines using real-time qPCR analysis. (**B**) The relative expression of *PC* in SKOV-3 cell after *PC* knockdown using shRNAs by real-time qPCR analysis. (**C**) Decreased expression of *PC* can significantly inhibit SKOV-3 cell proliferation. (**D**) Cell cycle analysis of SKOV-3 cells following *PC* knockdown. The effect of PC on cell cycle progression was examined using flow cytometry. *PC* knockdown significantly inhibited cell cycle transition from G1 to S phase. (**E**) Cell migration and invasion of ovarian cancer cells after *PC* knockdown. The effects of *PC* on migration and invasion of SKOV3 cells were evaluated using the Transwell system. PC knockdown significantly inhibited the invasive and metastatic abilities of ovarian tumor cells.

To evaluate the role of the metabolic pathway-related genes in ovarian cancer progression, we initially conducted real-time qPCR analyses to examine the expression levels of genes in different ovarian tumor cells. The results showed that *PC* was significantly upregulated in four cancer cell lines (SKOV3, CAOV-3, OV-1063, and OVCAR-3) compared to that in control IOSE80 cells (Figure 3A). Other genes were dysregulated in the different cell types. For example, *GGPS1* was upregulated in the OV-1063 and OVCAR-3 cells and downregulated in CAOV-3 cells; PPOX was only upregulated in OV-1063 and OVCAR-3 cells. Considering these findings, the siRNA knockdown of *PC* was designed to explore its roles in ovarian cancer development. The knockdown of *PC* was confirmed using qRT-PCR analysis, which revealed that the expression of *PC* was significantly reduced in SKOV3 cells-transfected with shPC-1 and shPC-3, and shPC-2 presented off-target effects (Figure 3B). The degree of knockdown for shPC-1 and shPC-3 were 66.7% and 58%, respectively. Thus, shPC-1 transfected SKOV3 cells were used for subsequent experiments.

### PC significantly inhibits proliferation, cell cycle progression, and cell migration and invasion in SKOV3 cells

The CCK-8 assay was conducted to evaluate the changes in proliferation of SKOV3 cells after *PC* knockdown. Depletion of *PC* significantly decreased proliferation in the shPC group compared to that of the shNC group (Figure 3C). The effect of PC on cell cycle progression was also examined using flow cytometry. The percentage of cells in the G1 phase was increased from 49.56% in the control group to 68.91% in shPC cells. In contrast, the percentage of cells in the S phase was decreased from 32.41% in the control group to 17.31% in the shPC group (Figure 3D). These results indicate that *PC*-knockdown significantly inhibited cell cycle transition from the G1 to S stage.

Invasion and metastasis are major events in the progression of cancers. Therefore, we further investigated the effect of PC on ovarian cell migration and invasion using the Transwell system. Results revealed that decreased expression of *PC* inhibited the invasive and metastatic abilities of ovarian tumor cells (Figure 3E).

### Transcriptome sequencing reveals genes and pathways regulated by PC

**Figure 4:**
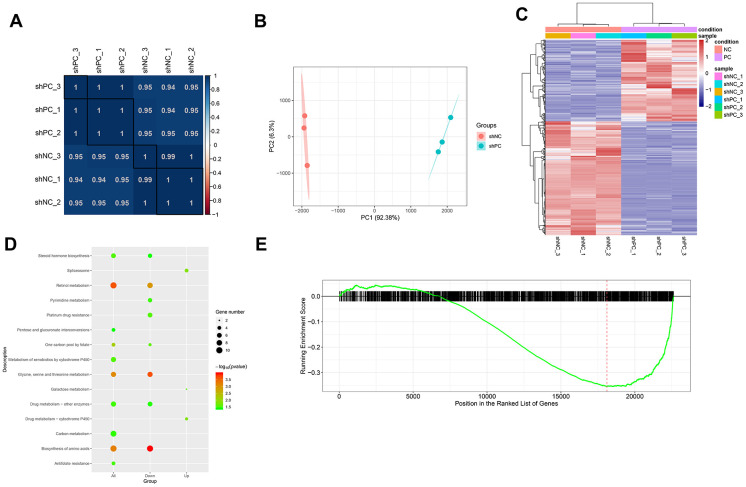
**Differentially expressed genes and pathways enrichment analysis after *PC* knockdown based on transcriptome sequencing.** (**A**) Sample correlation based on differential gene expression. The correlation between samples was analyzed using Pearson’s correlation coefficient based on gene expression values. There were significant positive correlations between samples. (**B**) Principal component analysis results. The different colored dots represent the sample group under the condition. (**C**) Heatmap of differentially expressed genes between shPC and shNC samples. Two-dimension clustering analysis results were visualized using heatmaps for differentially expressed genes from *PC*-knockdown samples compared to the normal control group. The gene expression profiles were significantly different between groups. Red represents high expression levels while blue represents low expression levels. (**D**) KEGG pathway enrichment analysis for differentially expressed genes. The top ten pathway terms ranked by p-value were visualized using dot plot. The vertical axis represents KEGG pathways and the horizontal axis shows differentially expressed genes. A category with a smaller p-value represents a more significant difference. (**E**) Gene set enrichment analysis results. The red line refers to the highest enrichment score.

After filtering out genes with low expression levels, a total of 22,838 gene expression matrices were obtained. The correlation between samples based on gene expression was measured using the Pearson’s correlation coefficient. A heat map of sample correlation is shown in Figure 4A. PCA analysis was performed for validation (Figure 4B) and the results showed that the samples in the different groups could be distinguished. Using thresholds of |log2FC| > 1.585 and FDR < 0.05, 1404 DEGs were identified in the shPC group compared to the shNC group, including 586 up-regulated genes and 818 down-regulated genes. Bidirectional clustering analysis illustrates that the expression profiles of DEGs are significantly different between the shPC and shNC groups (Figure 4C). We further analyzed the molecular functions of the genes and found that DEGs in shPC samples were mainly associated with steroid hormone biosynthesis and drug metabolism (Figure 4D). GSEA showed that the down-regulation of genes in the shPC group was more associated with pathogenesis of ovarian cancer compared to that in the shNC group (Figure 4E).

**Figure 5:**
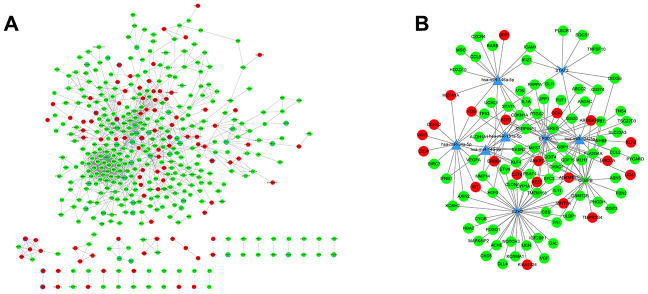
**PPI analysis and miRNA regulatory networks.** (**A**) PPI networks were constructed to visualize the relationships of differentially expressed genes screened from *PC*-knockdown samples compared to control samples. Red dots represent up-regulated genes and green dots refer to down-regulated genes. The points with a blue border refer to ovarian cancer-related genes. (**B**) miRNA regulatory network. A complex regulatory network was constructed to visualize connections of miRNAs, transcription factors, and genes related to ovarian cancer. The dots in red and green represent up- and down-regulated genes, respectively. Triangular nodes refer to miRNAs and v-shaped nodes represent transcription factors.

A PPI network was constructed, which consisted of 1495 edges connecting 431 nodes (Figure 5A). In the PPI network, STAT1 (degree = 54) and TP53 (degree = 46) had higher degree values than other genes, which indicated the significant role of STAT1 and TP53 in ovarian cancer progression. In the miRNA-TF-gene network, several miRNAs and TFs were identified as hub factors associated with ovarian cancer. Hsa-miR-124-3p, EZH2, and EP300 were the significant nodes in the miRNA-TF-gene network (Figure 5B).

### Metabolomics sequencing reveals the dysregulated metabolic profile induced by PC knockdown

**Figure 6:**
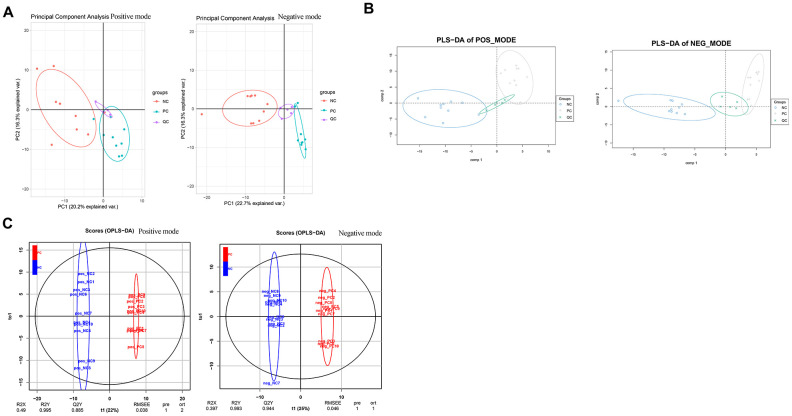
**Data normalization and partial least squares discriminant analysis for metabolomics data.** Two-dimensional principal component analysis (**A**) and three-dimensional partial least squares discriminant analysis (**B**) were conducted to ensure the detection stability of metabolomics data analysis. (**C**) Orthogonal partial least squares discriminant analysis by the X-Score model.

After normalization, the data were subjected to PCA and PLS-DA discriminant analyses under ESI+ and ESI- modes, separately. PCA showed that the samples in the different groups could be clustered together, and there were small offsets for the QC samples, which suggested data stability (Figure 6A). PLS-DA score plots showed obvious group separation between samples, indicating that the data were stable and reliable (Figure 6B). The cumulative R2Y and Q2Y values were both close to 1.0 (Figure 6C), which indicated that the PLS-DA models were stable, credible, and supported the differences between groups.

**Figure 7:**
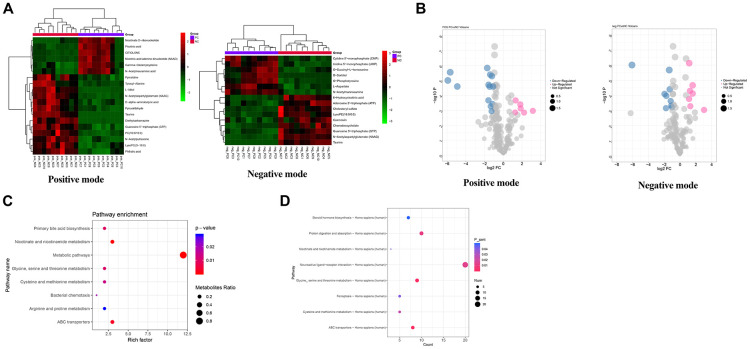
**Metabolomics data analysis for the identification of potential metabolic pathways regulated by PC.** (**A**) Heatmap of differential metabolites in shPC vs. shNC groups in positive mode and negative mode. A bidirectional clustering analysis heat map was used to visualize metabolite levels in shPC vs. shNC samples under positive (left) and negative modes (right). The horizontal and vertical axes represent the samples and metabolites, respectively. Green represents down-regulated levels while red refers to up-regulated levels. (**B**) Volcano map of differential metabolites under positive (left) and negative (right) modes in shPC vs. shNC samples. The points in pink, blue, and grey refer to metabolites with up, down, and normal regulated levels, respectively. The dot size represents the VIP value. (**C**) Functional enrichment analysis for differential metabolites. The vertical and horizontal axes represent pathway categories and count number, respectively. The dot size represents the metabolite ratio of pathway enrichment. The color changes from blue to red refer to decreasing p-values. A dot with a smaller p-value represents a more significant difference for the pathway category. (**D**) Integrated analysis of transcriptomic and metabolomics data to identify crucial pathways regulated by *PC*.

As for the shPC vs. shNC groups, a total of 44 metabolites met the cutoff value of FDR < 0.01, |log2FC| > 1, and VIP > 1, including 22 up-regulated and 22 down-regulated metabolites. Bidirectional clustering heat maps and volcano plots were used to visualize the results (Figure 7A and 7B). The differential metabolites in the shPC samples were mainly enriched in the metabolic pathways, ABC transporters, and nicotinate and niacinamide metabolism (Figure 7C).

### Dysregulated pathways at both transcriptomics and metabolomics levels

Using an integrated analysis of transcriptomic and metabolomics data, eight pathways were found to be dysregulated by DEGs and differential metabolites in SKOV3 cells induced by *PC* knockdown, such as neuroactive ligands receptor interaction; glycine, serine, and threonine metabolism; and ABC transporter pathways (Figure 7D).

## DISCUSSION

Ovarian cancer is one of the most common cancers in women and is a major cause of cancer-related death worldwide [16]. Although patients with ovarian cancer benefit from first-line treatments during early stages of cancer, most cases are diagnosed with a high-stage carcinoma. Despite advances in treatment management for ovarian cancer patients, the prognosis remains poor. The screening of potential biomarkers and a better understanding of the pathogenesis of ovarian cancer could contribute to the development of novel target therapies [17]. Thus, in this study, we attempted to discover prognosis-related biomarkers and explore the related mechanisms underlying the development and progression of ovarian cancer.

In our study, 66 prognosis-related genes in ovarian cancer samples were identified by combining TCGA and GSE49997 datasets. A metabolic pathway was the most significant pathway enriched by *GGPS1, ME1, DSE, NTPCR, PPOX*, and *PC.* *ME1* and *DSE* have been reported to be associated with the development of ovarian cancer in previous studies [18, 19]. Among the remaining metabolic pathway-related genes, the high expression of *PC* was closely related with poor survival of patients with ovarian cancer and *PC* was prominently overexpressed in the four ovarian cancer cell lines, making it a candidate gene for further analysis. To our knowledge, this is the first study exploring the clinical significance of *PC* in ovarian cancer.

Pyruvate carboxylase is an anaplerotic enzyme that catalyzes the irreversible carboxylation of pyruvate to oxaloacetate. It plays an essential role in various metabolic processes in mammals, such as gluconeogenesis, lipogenesis, biosynthesis of neurotransmitters, and glucose-induced insulin secretion [20]. A previous study shows that PC is significantly increased in gastric adenocarcinomas and correlates with tumor stage and pathological grade [21]. The expression and activity of PC is significantly enhanced in non-small cell lung cancer tissues and required for tumor cell proliferation [22]. A recent study by Phannasil et al. shows that the up-regulation of *PC* could promote cell growth and invasion of breast cancer cells [23]. In addition, the increased expression of PC is found in isocitrate dehydrogenase (IDH)-mutant glioma tissues and PC has been determined to be the therapeutic target [24]. Previous reports are consistent with findings of our *in vivo* experiments. Results of the present study show that cell proliferation, cell cycle progression, and cell invasion and migration were significantly inhibited in *PC-*depleted SKOV3 cells, which indicate, like its roles in other cancer types, that *PC* might function as a major tumor gene to promote the progression of ovarian cancer.

In order to further explore the changes in genes and metabolites in ovarian cancer cells induced by *PC* knockdown, transcriptome sequencing and metabolomics sequencing were performed. Our results showed that numerous genes and metabolites were differentially expressed in *PC*-knockdown SKOV3 cells.

The PPI network of DEGs induced by *PC* knockdown suggested that STAT1 (degree = 54) and TP53 (degree = 46) were the hub genes with high degrees, which revealed the significant role of these two proteins in the alteration of gene expression. STAT1 is a member of the STAT family and functions as a signal messenger involved in many biological processes, including cell proliferation, differentiation, and apoptosis. The activation of STAT1 is triggered by JAK ligands and increased expression levels of STAT1 have been reported in patients with ovarian cancer [25]. Overexpression or knockdown of STAT1 can directly induce or suppress ovarian cancer cell proliferation, migration, and invasion. One of the potential mechanisms might be related to crosstalk with TGF-β signaling pathways, which is a major factor in EOC progression [26]. In addition, TP53, also known as p53, is well-established as an important tumor suppressor. Mutations in p53 have been frequently found in human tumors including ovarian cancer. Novel functions of p53 in metabolic regulation have also been reported in recent studies [27, 28]. p53 is involved in the regulation of many processes such as glycolysis, mitochondrial metabolism, and fatty acid synthesis [29]. The analysis of the PPI network in the present study also demonstrated that STAT1 and p53 are candidate genes in ovarian cancer development, which is consistent with results from previous studies.

Additionally, metabolites are the final products in biological processes and can be influenced by genetic or environmental factors. Metabolomics analyses have demonstrated that pyruvate carboxylation acts as a key process, providing carbon backbones for downstream metabolites for the biosynthesis of cellular components such as membrane lipids, nucleotides, and amino acids [30]. By integrating the analysis of transcriptomic and metabolomics data, we demonstrated that these DEGs and differential metabolites in PC-knockdown tumor cells were closely associated with neuroactive ligands receptor interactions; glycine, serine, and threonine metabolism; and ABC transporter pathways. As described previously in the results, p53 plays an important role in the metabolic mechanisms of tumors. Mutations in p53 can enhance the glycolytic rate of fibroblasts and disturb biosynthetic processes of serine and glycine [27]. Furthermore, various ABC transporters were increased after EOC chemotherapy, which decreases the accumulation of chemotherapy drugs and finally induce a poor prognosis of ovarian cancer by enhancing chemo-resistance [31]. Considering this, we concluded that the disturbance of *PC* could significantly influence gene expression and metabolomics in ovarian cancer cells and lead to the dysregulation of serine, glycine biosynthesis, and ABC transporter pathways in ovarian cancer.

There are some limitations in our study. First, because of heterogeneity in the samples and technology limitations, the metabolites analyzed may be incomplete and not available for all ovarian cancer cases. Second, the sample size for the survival analysis was relatively small, and more samples should be included. Lastly, the function of *PC* was explored at the cellular level and functional validation in clinical samples is urgently needed.

## CONCLUSIONS

In conclusion, *PC* plays a significant role in the pathogenesis of ovarian cancer. The reduction of *PC* expression can significantly inhibit cell proliferation, cell cycle progression, and cell migration and invasion. Significantly decreased *PC* expression may impact ovarian tumor biology through the dysregulation of STAT1 and TP53 expression, serine and glycine biosynthesis, and ABC transporter pathways. The identified pathways in this study might provide new diagnostic and treatment options for patients with ovarian cancer.

## MATERIALS AND METHODS

### Data sources

The mRNA expression profiles associated with ovarian cancer were downloaded from The Cancer Genome Atlas (TCGA) database, which were derived from 299 tissue samples. The microarray dataset GSE49997 was downloaded from the Gene Expression Omnibus (GEO) repository (https://www.ncbi.nlm.nih.gov/geo/) based on the platform of GPL2986 ABI Human Genome Survey Microarray Version 2. The GSE49997 dataset includes 204 EOC tissue samples, of which 194 samples contain corresponding clinical information.

### Identification of survival-related genes

To identify crucial genes in ovarian cancer development, the gene expression profiles of samples with clinical information from TCGA and GSE49997 datasets were further analyzed. The raw count data were downloaded and normalized using the count per million (CPM) method. Genes with low expression levels were removed, and the remaining 15,683 genes with high expression levels were subjected to survival analyses. Prognostic risk assessment was based on survival information and normalized gene expression profiles. Patients were divided into high-expression and low-expression groups based on the median value of gene expression. Kaplan-Meier (KM) curves were generated using the Survival package in R and statistically tested using the log-rank test. Genes with a p-value of less than 0.05 were considered as potential genes associated with prognosis in EOC patients. Similarly, after normalization, the gene expression datasets from EOC tissues in the GEO dataset were subjected to survival analyses. The genes closely related with prognosis based on the two databases were analyzed using Venn diagrams. The overlapping genes were then selected for further analysis.

### Pathway enrichment analysis and literature search

Text mining was performed for the overlapping genes using Perl code. The published genes that are closely related with ovarian cancer were searched in the PubMed database. In addition, the overlapping genes were subjected to the Kyoto Encyclopedia of Genes and Genomes (KEGG) pathway analysis using the Database for Annotation, Visualization and Integrated Discovery (DAVID) online tool. Pathways with p < 0.05 and counts ≥ 2 were considered significant.

### Cell culture

Four human ovarian cancer cell lines (SKOV3, Caov-3, OV-1063, OVCAR-3), normal epithelial ovarian cells (IOSE80), and human embryonic kidney (293T) cells were purchased from the cell bank of China Academic of Science. The SKOV3 cells were cultured in McCoy’s 5A Media (modified with tricine) supplemented with 10% fetal bovine serum (FBS). The Caov-3 and 293T cell lines were maintained in 90% DMEM with 10% FBS. The OV-1063 and IOSE80 cell lines were maintained in 90% RPMI 1640 with 10% FBS, and the OVCAR-3 cells were cultured in 80% RPMI 1640 with 20% FBS, sodium pyruvate, and 0.01 mg/ml bovine insulin. All the cell lines were cultured in an atmosphere of 5% CO_2_ and 95% air at 37 °C.

### Real-time qPCR analysis

Total RNA was extracted from cells using the RNAiso Plus (Trizol) reagent (TaKaRa, Japan) and cDNA was synthesized using the PrimeScript™RT Master Mix (Perfect Real Time) kit (TAKARA, Japan) according to the manufacturer’s instructions. Real-time PCR was performed to evaluate the expression levels of *GGPS1*, *NTPCR*, *PPOX*, *PC*, *PRICKLE2*, *TCF7L1*, and *PPP3CA* in tumor cells. A total of 8 μl of cDNA was used as template in a final 20 μl PCR volume containing 1 μl forward primer, 1 μl reverse primer, and 10 μl SYBR Premix EX Taq (2x). PCRs were run as follows: 50.0 °C for 3 min, 95.0 °C for 3 min, followed by 40 cycles of 95.0 °C for 10 s and 60.0 °C for 30 s. Following PCR, a melting curve was obtained at temperatures from 60 °C to 95 °C, at increments of 0.5 °C for 10 s. Primer sequences are listed in Table 3.

**Table 3:** The primer sequences in PCR analysis.

**Symbol**	**Sequences (5’-3)**
GGPS1-hF	CTGCGTGGACCGATTAGCTTT
GGPS1-hR	TCTGTAGCTTGTCCTCTGGAAC
NTPCR-hF	ACCCGTCTTGAGGAATGTGA
NTPCR-hR	CTCTTGAACTGGGCACTCCT
PPOX-hF	GGACTGAAGGAGATGCCGAG
PPOX-hR	CAACCTGTGAGCAGTCAGGA
PC-hF	GACGGCGAGGAGATAGTGTC
PC-hR	TGGCAATCTCACCTCTGTTGG
PRICKLE2-hF	GTCTGTTGCCAGCTTCAGGA
PRICKLE2-hR	TCACTGTCACCATGTGCTCC
TCF7L1-hF	TCCAAAGACAGGAATCCCCCG
TCF7L1-hR	TGAGGAGAGAACCGACTGGA
PPP3CA-hF	CGGGGTGTGCAGTCGG
PPP3CA-hR	TTTGCTGTAAGCCGGTGACT
shPC-1-F	CCGGGCCCAGTTTATGGTGCAGAATTTCAAGAGAATTCTGCACCATAAACTGGGCTTTTTTGGTACC
shPC-1-R	AATTGGTACCAAAAAAGCCCAGTTTATGGTGCAGAATTCTCTTGAAATTCTGCACCATAAACTGGGC
shPC-2-F	CCGGGCCAAGGAGAACAACGTAGATTTCAAGAGAATCTACGTTGTTCTCCTTGGCTTTTTTGGTACC
shPC-2-R	AATTGGTACCAAAAAAGCCAAGGAGAACAACGTAGATTCTCTTGAAATCTACGTTGTTCTCCTTGGC
shPC-3-F	CCGGATGGGCATCCGCCTGGATAATTTCAAGAGAATTATCCAGGCGGATGCCCAT TTTTTTGGTACC
shPC-3-R	AATTGGTACCAAAAAAATGGGCATCCGCCTGGATAATTCTCTTGAAATTATCCAGGCGGATGCCCAT
shNC-F	CCGGGTTCTCCGAACGTGTCACGTCAAGAGATTACGTGACACGTTCGGAGAATCTTTTGGTACC
shNC-R	AATTGGTACCAAAAGATTCTCCGAACGTGTCACGTAATCTCTTGACGTGACACGTTCGGAGAAC
GAPDH-hF	TGACAACTTTGGTATCGTGGAAGG
GAPDH-hR	AGGCAGGGATGATGTTCTGGAGAG

### Cell transfections

Based on designing sigma software (https://www.sigmaaldrich.com/life-science/functional-genomics-and-rnai/sirna/mission-predesigned-sirna.html), the shRNA sequence of *PC* was obtained. Three shRNAs sequences in CDS region for *PC*, GCCCAGTTTATGGTGCAGAAT (shPC-1), GCCAAGGAGAACAACGTAGAT (shPC-2), and ATGGGCATCCGCCTGGATAAT (shPC-3) were selected to knock down endogenous *PC*, and messy sequence (NC- GTTCTCCGAACGTGTCACGTC) was used as control sequence. Then, the primers were designed and synthesized by Suzhou Jinweizhi Biological Technology Co. LTD. (Table 3). Inducible shRNA construction was performed by ligating annealed oligonucleotides into the pLKO.1 Puro vector digested with EcoRI and AgeI. The ligated products were transformed into chemically competent Stbl3 *E. coli* using Lipofectamine 3000 reagent (Invitrogen) according to the manufacturer’s instructions and grown in LB supplemented with ampicillin. After validation of shRNA insertion using gene sequencing, the plasmids were packaged into lentiviral particles using the third-generation lentiviral production system. SKOV3 cells were divided into three groups: black (without any treatment), shNC (transfected with negative control plasmid), shPC (transfected with shRNA-PC). Stable SKOV3 cells were selected with 0.5 μg/ml of puromycin over 3 days. Lastly, the efficiency of *PC* knockdown in SKOV3 cells was confirmed using real-time qPCR analysis, and the degree of knockdown [(1- the relative mean expression of *PC* in shPC group/shNC group) × 100%] for three *PC* shRNAs were calculated. The sable shRNA*-PC* transfected SKOV3 cells with highest knockdown degree were used for following experiments.

### Cell viability and cell cycle analyses

Cell viability assays were performed using the cell counting kit-8 (CCK-8; Beyotime Biotechnology, China). Briefly, the SKOV3 cells in three groups (3 × 10^4^ cells/ml) were seeded in 96-well plates at a density of 3,000 cells/well, respectively. Cells were incubated at 37 °C in 5% CO_2_ atmosphere conditions for 24, 48, 72, and 96 hours, and then 10 μl of CCK-8 solution was added into each well for 1.5 hours. The OD value of each well sample was measured at 450 nm using the Multiscan Spectrum System (BD Biosciences, USA). All assays were repeated in triplicate.

Flow cytometry was used for cell cycle analyses. shPC and control SKOV3 cells were digested with trypsin and collected using centrifugation at 300 × *g*, 4 °C for 5 min. Then, cells were fixed in 70% ethanol overnight at 4 °C. Subsequently, the samples were treated with RNase A for 30 min and stained with propidium iodide (BD Biosciences, USA) for 15 min. Flow cytometry was immediately conducted using a FACSCAN flow cytometer (BD Biosciences, USA) following the manufacturer’s instructions. All assays were repeated in triplicate.

### Transwell migration and invasion assays

Invasion and migration assays were performed using BD Matrigel culture inserts. First, 8 μm pore 24-well Transwell inserts (Corning, USA) were coated with 20 μl of Matrigel (BD Biosciences) and incubated for 30 min at 37 °C in a 5% CO_2_ incubator to allow gel formation. Control shNC and shPC SKOV3 cells were suspended at equal cell densities in serum-free medium. A total of 100 μl of cells (2 × 10^5^ cells/ml) were seeded in the upper chamber of untreated Transwells for cell migration analyses and in the upper chamber of Transwells treated with Matrigel for cell invasion analyses. The lower chambers were supplemented with 500 μl of 10% FBS-containing medium. Then, cells were incubated at 37 °C in 5% CO_2_ atmosphere conditions for 12 and 24 h, respectively. The cells in Transwells were treated with 4% paraformaldehyde for 30 min and stained with crystal violet for 10 min. Subsequently, the upper chambers were washed three times in PBS and non-invading cells on the inner surface were carefully removed using cotton swabs. Finally, the invading cells on the reverse side of the upper chamber were counted using a microscope at three different fields to assess cell migration and invasion abilities. All assays were repeated in triplicate.

### Metabolomics data acquisition and identification

Metabolomics data acquisition was performed using an ultra-performance liquid chromatography-quadrupole-time of flight liquid chromatography/mass spectrometry (UPLC-Q-TOF LC/MS) system [32]. The samples included 10 shPC-infected SKOV3 cell samples (1 × 10^7^ cells for each sample), 10 shNC SKOV3 cell samples, and 5 quality control (QC) samples. Cells in each sample were mixed with 500 μl of methyl alcohol/acetonitrile/distilled water (2:2:1, v/v/v) solution and disrupted using ultrasonication for 30 min. After incubation at -20 °C for 1 h, the supernatant was collected using centrifugation at 13,000 rpm at 4 °C for 15 min followed by freeze-drying. For metabolomic analyses, samples were re-dissolved in 100 μl of acetonitrile solution (1:1 ratio of acetonitrile and water, by volume) and centrifuged at 14,000 × *g* at 4 °C for 15 min. The supernatant was collected for LC/MS analysis.

The metabolic products acquisition and identification were both performed in positive-ion (ESI+) and negative-ion (ESI-) modes and analyzed using a Triple-TOF 5600 mass spectrometer. Data normalizations were performed on LC/MS data based on internal standards using ESI+ and ESI- modes.

Principal component analysis (PCA), extensively used in the statistical learning field, and the partial least squares discriminant analysis (PLS-DA) are commonly applied to evaluate differences between groups [33, 34]. In the present study, we used two-dimensional PCA to ensure detection stability and three-dimensional PLS-DA for cross validation. In addition, univariate nonparametric analyses and multivariate PLS-DA were performed for metabolic profiling using the ropls software, version 1.6.2 (http://bioconductor.org/packages/release/bioc/html/ropls.html). The thresholds for potential biomarker selection were set as follows: a false discovery rate (FDR) of <0.01, |log2 fold change (log2FC)| > 1 in univariate analysis, and Variable Importance in the Projection (VIP) > 1 in multivariate analysis. Bidirectional hierarchical clustering analyses were performed to assess data classification ability and concentration levels of the screened metabolites. For differential metabolites obtained from tumor cells, we transformed these data into the KEGG ID format using the MetaboAnalyst online tool [35] and performed KEGG pathway analyses using MBROLE 2.0 (http://csbg.cnb.csic.es/mbrole2/analysis.php) with a p-value < 0.05.

### Transcriptome sequencing

Total mRNA extractions and cDNA library preparations were performed for shPC and control shNC cell samples. Then, two-paired end sequencing was performed using the Illumina platform (Illumina, San Diego, CA, USA).

Clean reads data were obtained using the Trimmomatic tool (version 3.6) [36]. Then, the reads were mapped to the human reference genome (GRCH38, Gencode) [37] with the Hisat 2 software, version 2.05 [38]. Gene expression levels were evaluated by counting reads mapped to protein-coding regions using FeatureCounts tools (v1.6.0) [39], and expression values were normalized using Fragments Per Kilobase of Exon Per Million Fragments Mapped (FPKM) method. Genes with an FPKM value ≥ 0.1 in at least three samples were further analyzed. According to filtered gene abundance expression profiles, the correlation of gene expression levels between samples was analyzed using PCA with the ggord package (https://zenodo.org/badge/latestdoi/35334615) [40].

DEGs between the shPC and shNC groups were identified using the quasi-likelihood F-tests method of the edgeR software [41]. Genes with |log2FC| > 1.585 and FDR < 0.05 were considered to be significant, and two-dimensional clustering heatmaps were used to visualize the gene expression profiles of DEGs. Subsequently, gene ontology (GO) function and KEGG pathway enrichments were performed for genes of interest using the Clusterprofiler package [42]. A p-value < 0.05 was considered as a significant difference.

Ovarian carcinoma-related genes were retrieved from the DisGeNET database [43] (http://www.disgenet.org/web/DisGeNET/menu/home). Differentially expressed genes were subjected to gene set enrichment analysis (GSEA) using the DOSE package [44].

The PPI pairs with required confidence (combined score > 0.7) were obtained with the STRING online tool [45] (https://string-db.org/). Cytoscape software [46] was utilized to construct a PPI network and the network topological properties were analyzed based on degree [47], betweenness [48], and closeness [49] using the cytoscape CytoNCA plugin [50].

Moreover, the significant modules in the PPI network were mined using the MCODE tool [51] with a screening score > 10. For the intriguing DEGs, we performed microRNA and transcription factor (TF) predictions by using Enrichr tools [52]. The corresponding miRNA-gene pairs and TF-gene pairs were also identified from the miRTarBase and ENCODE databases [53]. Finally, we integrated these miRNAs, TFs, and candidate genes to construct a systemic regulatory network.

### Integrated pathway analysis of transcriptomics and metabolomics data

Integrated analyses are conducted in order to understand the biological function of post-genomic data at a higher level than individual biomolecules. The “IMPaLA” web tool has been used to integrate more than one type of omics data for pathway analysis [54]. In this study, the joint pathway analysis was performed for DEGs and differential metabolites based on the KEGG database. Lastly, pathways with number_of_overlapping_metabolites/gene > 0 and metabolite with p < 0.05 were considered to be significant.
